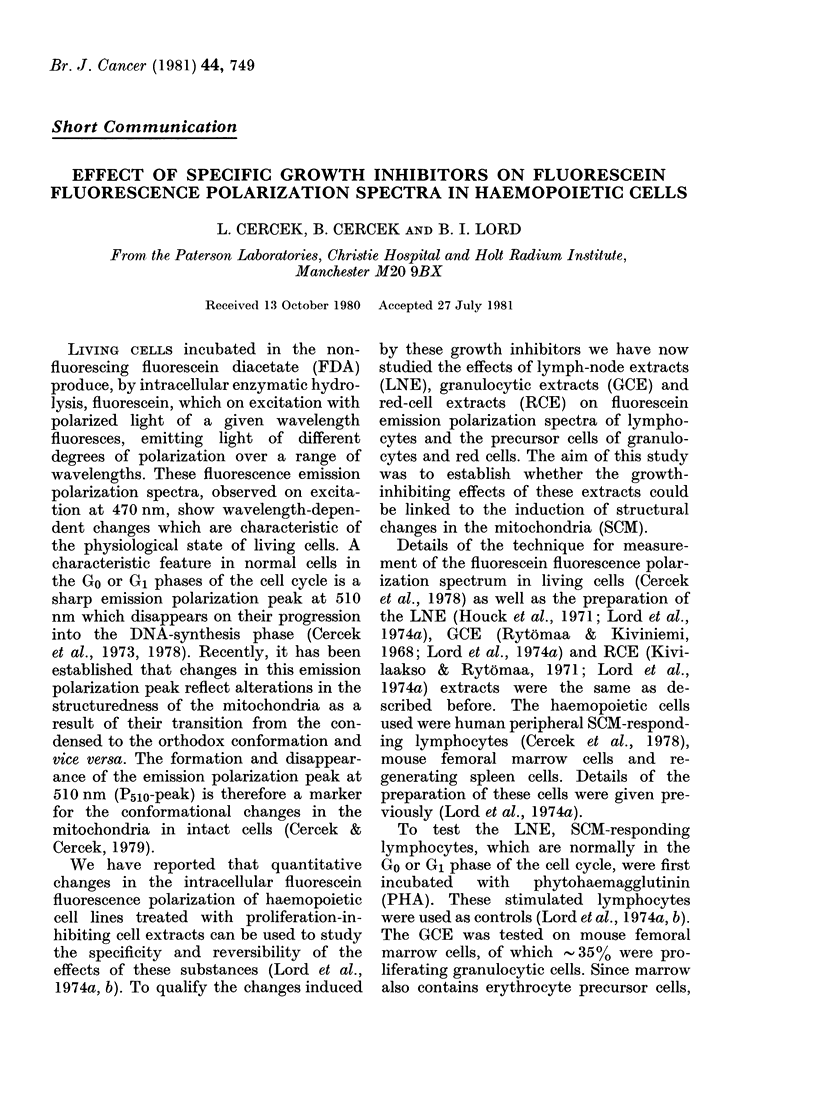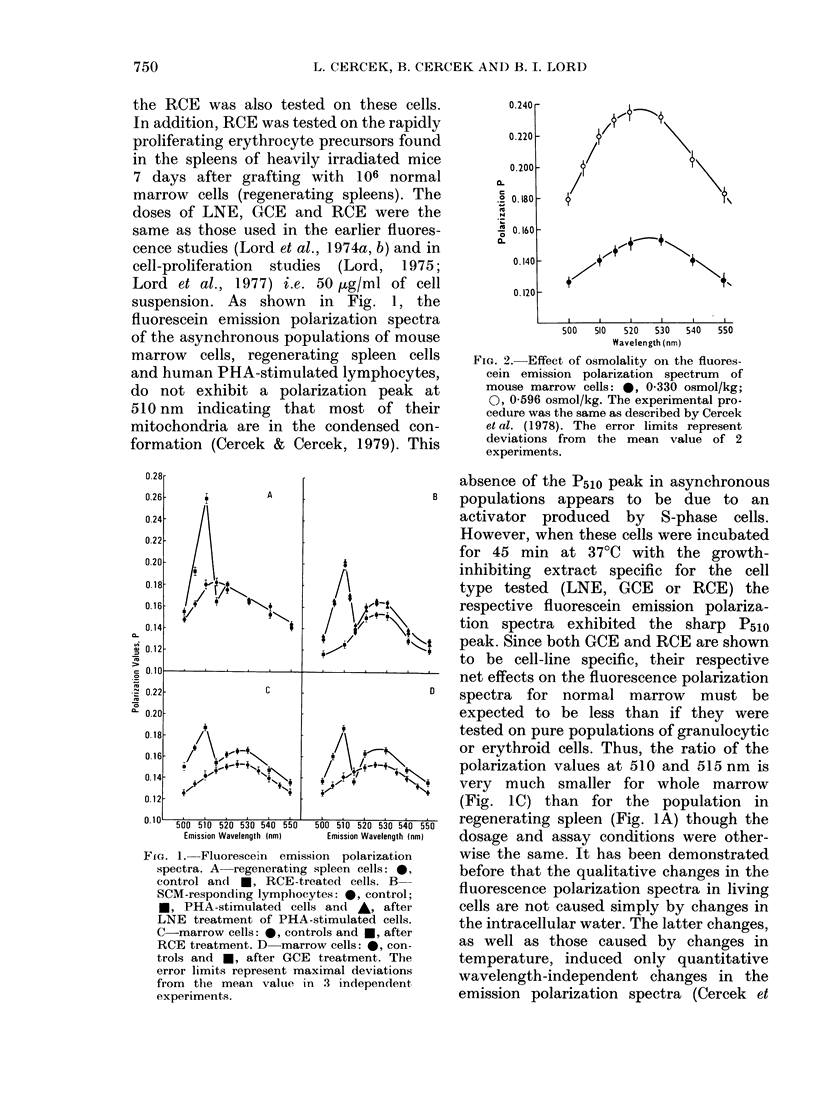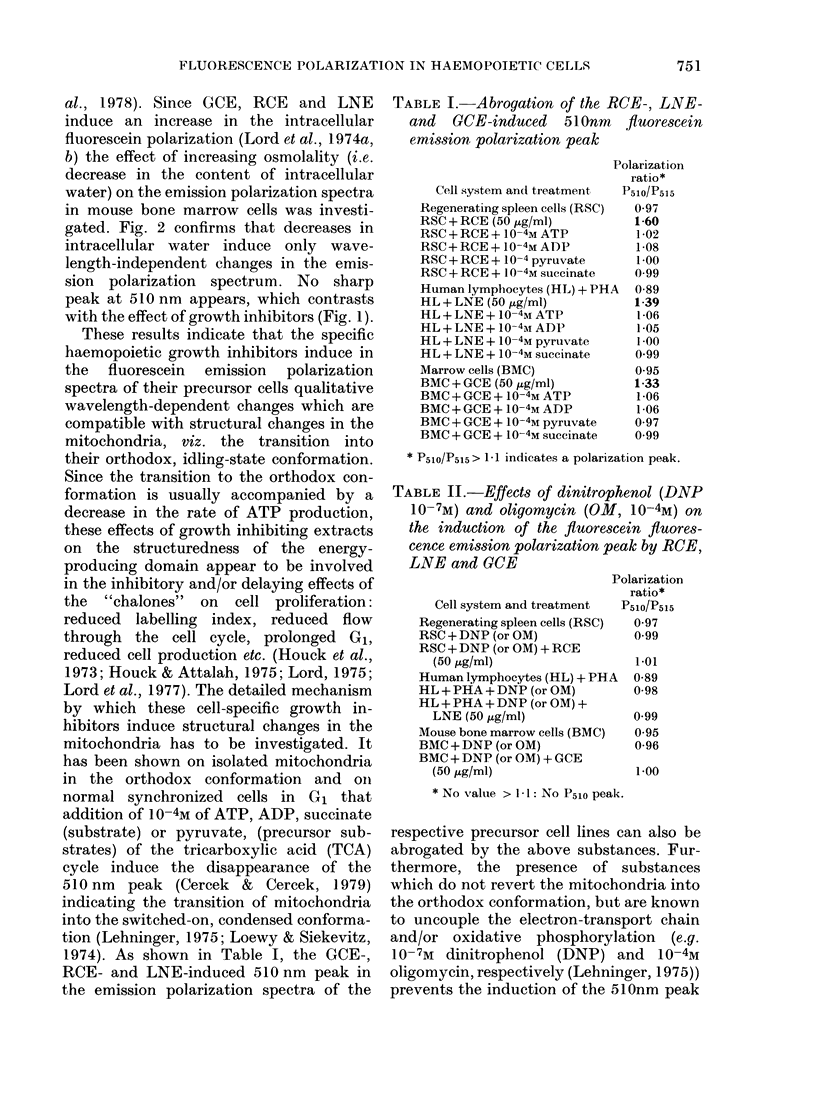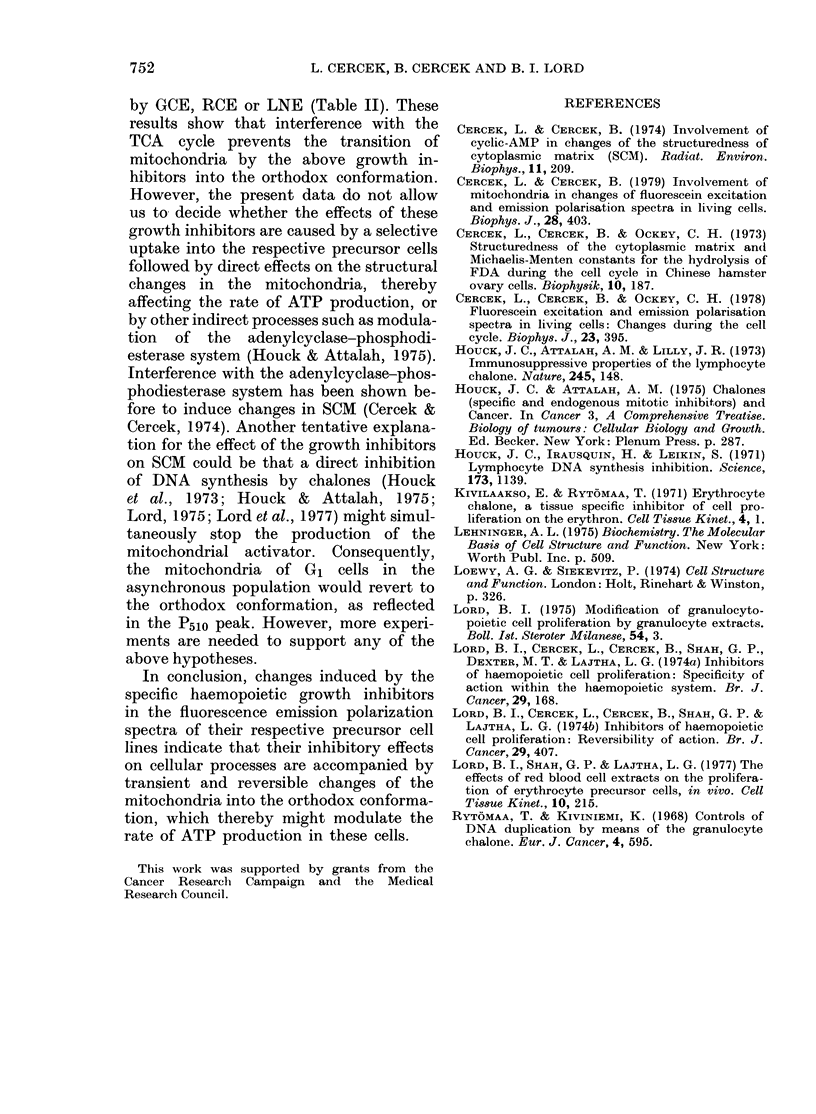# Effect of specific growth inhibitors on fluorescein fluorescence polarization spectra in haemopoietic cells.

**DOI:** 10.1038/bjc.1981.263

**Published:** 1981-11

**Authors:** L. Cercek, B. Cercek, B. I. Lord


*Br. J. Cancer* (1981) **44**, 749

***Short Communication***

### EFFECT OF SPECIFIC GROWTH INHIBITORS ON FLUORESCEIN FLUORESCENCE POLARIZATION SPECTRA IN HAEMOPOIETIC CELLS

L. CERCEK, B. CERCEK AND B. I. LORD

*From the Paterson Laboratories, Christie Hospital and Holt Radium Institute, Manchester M20 9BX*

Received 13 October 1980 Accepted 27 July 1981

LIVING CELLS incubated in the non-fluorescing fluorescein diacetate (FDA) produce, by intracellular enzymatic hydrolysis, fluorescein, which on excitation with polarized light of a given wavelength fluoresces, emitting light of different degrees of polarization over a range of wavelengths. These fluorescence emission polarization spectra, observed on excitation at 470 nm, show wavelength-dependent changes which are characteristic of the physiological state of living cells. A characteristic feature in normal cells in the $G_0$ or $G_1$ phases of the cell cycle is a sharp emission polarization peak at 510 nm which disappears on their progression into the DNA-synthesis phase (Cercek *et al.*, 1973, 1978). Recently, it has been established that changes in this emission polarization peak reflect alterations in the structuredness of the mitochondria as a result of their transition from the condensed to the orthodox conformation and *vice versa*. The formation and disappearance of the emission polarization peak at 510 nm ($P_{510}$-peak) is therefore a marker for the conformational changes in the mitochondria in intact cells (Cercek & Cercek, 1979).

We have reported that quantitative changes in the intracellular fluorescein fluorescence polarization of haemopoietic cell lines treated with proliferation-inhibiting cell extracts can be used to study the specificity and reversibility of the effects of these substances (Lord *et al.*, 1974*a*, *b*). To qualify the changes induced by these growth inhibitors we have now studied the effects of lymph-node extracts (LNE), granulocytic extracts (GCE) and red-cell extracts (RCE) on fluorescein emission polarization spectra of lymphocytes and the precursor cells of granulocytes and red cells. The aim of this study was to establish whether the growth-inhibiting effects of these extracts could be linked to the induction of structural changes in the mitochondria (SCM).

Details of the technique for measurement of the fluorescein fluorescence polarization spectrum in living cells (Cercek *et al.*, 1978) as well as the preparation of the LNE (Houck *et al.*, 1971; Lord *et al.*, 1974*a*), GCE (Rytömaa & Kiviniemi, 1968; Lord *et al.*, 1974*a*) and RCE (Kivilaakso & Rytömaa, 1971; Lord *et al.*, 1974*a*) extracts were the same as described before. The haemopoietic cells used were human peripheral SCM-responding lymphocytes (Cercek *et al.*, 1978), mouse femoral marrow cells and regenerating spleen cells. Details of the preparation of these cells were given previously (Lord *et al.*, 1974*a*).

To test the LNE, SCM-responding lymphocytes, which are normally in the $G_0$ or $G_1$ phase of the cell cycle, were first incubated with phytohaemagglutinin (PHA). These stimulated lymphocytes were used as controls (Lord *et al.*, 1974*a*, *b*). The GCE was tested on mouse femoral marrow cells, of which ~35% were proliferating granulocytic cells. Since marrow also contains erythrocyte precursor cells, the RCE was also tested on these cells. In addition, RCE was tested on the rapidly proliferating erythrocyte precursors found in the spleens of heavily irradiated mice 7 days after grafting with $10^6$ normal marrow cells (regenerating spleens). The doses of LNE, GCE and RCE were the same as those used in the earlier fluorescence studies (Lord *et al.*, 1974*a*, *b*) and in cell-proliferation studies (Lord, 1975; Lord *et al.*, 1977) *i.e.* 50 $\mu$g/ml of cell suspension. As shown in Fig. 1, the fluorescein emission polarization spectra of the asynchronous populations of mouse marrow cells, regenerating spleen cells and human PHA-stimulated lymphocytes, do not exhibit a polarization peak at 510 nm indicating that most of their mitochondria are in the condensed conformation (Cercek & Cercek, 1979). This

FIG. 2.—Effect of osmolality on the fluorescein emission polarization spectrum of mouse marrow cells: ●, 0·330 osmol/kg; ○, 0·596 osmol/kg. The experimental procedure was the same as described by Cercek *et al.* (1978). The error limits represent deviations from the mean value of 2 experiments.

FIG. 1.—Fluorescein emission polarization spectra. A—regenerating spleen cells: ●, control and ■, RCE-treated cells. B—SCM-responding lymphocytes: ●, control; ■, PHA-stimulated cells and ▲, after LNE treatment of PHA-stimulated cells. C—marrow cells: ●, controls and ■, after RCE treatment. D—marrow cells: ●, controls and ■, after GCE treatment. The error limits represent maximal deviations from the mean value in 3 independent experiments.

absence of the $P_{510}$ peak in asynchronous populations appears to be due to an activator produced by S-phase cells. However, when these cells were incubated for 45 min at 37°C with the growth-inhibiting extract specific for the cell type tested (LNE, GCE or RCE) the respective fluorescein emission polarization spectra exhibited the sharp $P_{510}$ peak. Since both GCE and RCE are shown to be cell-line specific, their respective net effects on the fluorescence polarization spectra for normal marrow must be expected to be less than if they were tested on pure populations of granulocytic or erythroid cells. Thus, the ratio of the polarization values at 510 and 515 nm is very much smaller for whole marrow (Fig. 1C) than for the population in regenerating spleen (Fig. 1A) though the dosage and assay conditions were otherwise the same. It has been demonstrated before that the qualitative changes in the fluorescence polarization spectra in living cells are not caused simply by changes in the intracellular water. The latter changes, as well as those caused by changes in temperature, induced only quantitative wavelength-independent changes in the emission polarization spectra (Cercek *et*

*al.*, 1978). Since GCE, RCE and LNE induce an increase in the intracellular fluorescein polarization (Lord *et al.*, 1974*a*, *b*) the effect of increasing osmolality (*i.e.* decrease in the content of intracellular water) on the emission polarization spectra in mouse bone marrow cells was investigated. Fig. 2 confirms that decreases in intracellular water induce only wavelength-independent changes in the emission polarization spectrum. No sharp peak at 510 nm appears, which contrasts with the effect of growth inhibitors (Fig. 1).

These results indicate that the specific haemopoietic growth inhibitors induce in the fluorescein emission polarization spectra of their precursor cells qualitative wavelength-dependent changes which are compatible with structural changes in the mitochondria, *viz.* the transition into their orthodox, idling-state conformation. Since the transition to the orthodox conformation is usually accompanied by a decrease in the rate of ATP production, these effects of growth inhibiting extracts on the structuredness of the energy-producing domain appear to be involved in the inhibitory and/or delaying effects of the "chalones" on cell proliferation: reduced labelling index, reduced flow through the cell cycle, prolonged $G_1$, reduced cell production *etc.* (Houck *et al.*, 1973; Houck & Attalah, 1975; Lord, 1975; Lord *et al.*, 1977). The detailed mechanism by which these cell-specific growth inhibitors induce structural changes in the mitochondria has to be investigated. It has been shown on isolated mitochondria in the orthodox conformation and on normal synchronized cells in $G_1$ that addition of $10^{-4}$M of ATP, ADP, succinate (substrate) or pyruvate, (precursor substrates) of the tricarboxylic acid (TCA) cycle induce the disappearance of the 510 nm peak (Cercek & Cercek, 1979) indicating the transition of mitochondria into the switched-on, condensed conformation (Lehninger, 1975; Loewy & Siekevitz, 1974). As shown in Table I, the GCE-, RCE- and LNE-induced 510 nm peak in the emission polarization spectra of the respective precursor cell lines can also be abrogated by the above substances. Furthermore, the presence of substances which do not revert the mitochondria into the orthodox conformation, but are known to uncouple the electron-transport chain and/or oxidative phosphorylation (*e.g.* $10^{-7}$M dinitrophenol (DNP) and $10^{-4}$M oligomycin, respectively (Lehninger, 1975)) prevents the induction of the 510nm peak

TABLE I.—*Abrogation of the RCE-, LNE- and GCE-induced 510nm fluorescein emission polarization peak*

| Cell system and treatment | Polarization ratio* $P_{510}/P_{515}$ |
|---|---|
| Regenerating spleen cells (RSC) | 0·97 |
| RSC + RCE (50 μg/ml) | **1·60** |
| RSC + RCE + $10^{-4}$M ATP | 1·02 |
| RSC + RCE + $10^{-4}$M ADP | 1·08 |
| RSC + RCE + $10^{-4}$ pyruvate | 1·00 |
| RSC + RCE + $10^{-4}$M succinate | 0·99 |
| Human lymphocytes (HL) + PHA | 0·89 |
| HL + LNE (50 μg/ml) | **1·39** |
| HL + LNE + $10^{-4}$M ATP | 1·06 |
| HL + LNE + $10^{-4}$M ADP | 1·05 |
| HL + LNE + $10^{-4}$M pyruvate | 1·00 |
| HL + LNE + $10^{-4}$M succinate | 0·99 |
| Marrow cells (BMC) | 0·95 |
| BMC + GCE (50 μg/ml) | **1·33** |
| BMC + GCE + $10^{-4}$M ATP | 1·06 |
| BMC + GCE + $10^{-4}$M ADP | 1·06 |
| BMC + GCE + $10^{-4}$M pyruvate | 0·97 |
| BMC + GCE + $10^{-4}$M succinate | 0·99 |

* $P_{510}/P_{515} > 1·1$ indicates a polarization peak.

TABLE II.—*Effects of dinitrophenol (DNP* $10^{-7}$M) *and oligomycin (OM,* $10^{-4}$M) *on the induction of the fluorescein fluorescence emission polarization peak by RCE, LNE and GCE*

| Cell system and treatment | Polarization ratio* $P_{510}/P_{515}$ |
|---|---|
| Regenerating spleen cells (RSC) | 0·97 |
| RSC + DNP (or OM) | 0·99 |
| RSC + DNP (or OM) + RCE (50 μg/ml) | 1·01 |
| Human lymphocytes (HL) + PHA | 0·89 |
| HL + PHA + DNP (or OM) | 0·98 |
| HL + PHA + DNP (or OM) + LNE (50 μg/ml) | 0·99 |
| Mouse bone marrow cells (BMC) | 0·95 |
| BMC + DNP (or OM) | 0·96 |
| BMC + DNP (or OM) + GCE (50 μg/ml) | 1·00 |

* No value > 1·1: No $P_{510}$ peak.

by GCE, RCE or LNE (Table II). These results show that interference with the TCA cycle prevents the transition of mitochondria by the above growth inhibitors into the orthodox conformation. However, the present data do not allow us to decide whether the effects of these growth inhibitors are caused by a selective uptake into the respective precursor cells followed by direct effects on the structural changes in the mitochondria, thereby affecting the rate of ATP production, or by other indirect processes such as modulation of the adenylcyclase–phosphodiesterase system (Houck & Attalah, 1975). Interference with the adenylcyclase–phosphodiesterase system has been shown before to induce changes in SCM (Cercek & Cercek, 1974). Another tentative explanation for the effect of the growth inhibitors on SCM could be that a direct inhibition of DNA synthesis by chalones (Houck *et al.*, 1973; Houck & Attalah, 1975; Lord, 1975; Lord *et al.*, 1977) might simultaneously stop the production of the mitochondrial activator. Consequently, the mitochondria of $G_1$ cells in the asynchronous population would revert to the orthodox conformation, as reflected in the $P_{510}$ peak. However, more experiments are needed to support any of the above hypotheses.

In conclusion, changes induced by the specific haemopoietic growth inhibitors in the fluorescence emission polarization spectra of their respective precursor cell lines indicate that their inhibitory effects on cellular processes are accompanied by transient and reversible changes of the mitochondria into the orthodox conformation, which thereby might modulate the rate of ATP production in these cells.

This work was supported by grants from the Cancer Research Campaign and the Medical Research Council.

REFERENCES

Cercek, L. & Cercek, B. (1974) Involvement of cyclic-AMP in changes of the structuredness of cytoplasmic matrix (SCM). *Radiat. Environ. Biophys.*, **11**, 209.

Cercek, L. & Cercek, B. (1979) Involvement of mitochondria in changes of fluorescein excitation and emission polarisation spectra in living cells. *Biophys. J.*, **28**, 403.

Cercek, L., Cercek, B. & Ockey, C. H. (1973) Structuredness of the cytoplasmic matrix and Michaelis-Menten constants for the hydrolysis of FDA during the cell cycle in Chinese hamster ovary cells. *Biophysik*, **10**, 187.

Cercek, L., Cercek, B. & Ockey, C. H. (1978) Fluorescein excitation and emission polarisation spectra in living cells: Changes during the cell cycle. *Biophys. J.*, **23**, 395.

Houck, J. C., Attalah, A. M. & Lilly, J. R. (1973) Immunosuppressive properties of the lymphocyte chalone. *Nature*, **245**, 148.

Houck, J. C. & Attalah, A. M. (1975) Chalones (specific and endogenous mitotic inhibitors) and Cancer. In *Cancer* 3, *A Comprehensive Treatise. Biology of tumours: Cellular Biology and Growth.* Ed. Becker. New York: Plenum Press. p. 287.

Houck, J. C., Irausquin, H. & Leikin, S. (1971) Lymphocyte DNA synthesis inhibition. *Science*, **173**, 1139.

Kivilaakso, E. & Rytömaa, T. (1971) Erythrocyte chalone, a tissue specific inhibitor of cell proliferation on the erythron. *Cell Tissue Kinet.*, **4**, 1.

Lehninger, A. L. (1975) *Biochemistry. The Molecular Basis of Cell Structure and Function.* New York: Worth Publ. Inc. p. 509.

Loewy, A. G. & Siekevitz, P. (1974) *Cell Structure and Function.* London: Holt, Rinehart & Winston, p. 326.

Lord, B. I. (1975) Modification of granulocytopoietic cell proliferation by granulocyte extracts. *Boll. Ist. Steroter Milanese*, **54**, 3.

Lord, B. I., Cercek, L., Cercek, B., Shah, G. P., Dexter, M. T. & Lajtha, L. G. (1974*a*) Inhibitors of haemopoietic cell proliferation: Specificity of action within the haemopoietic system. *Br. J. Cancer*, **29**, 168.

Lord, B. I., Cercek, L., Cercek, B., Shah, G. P. & Lajtha, L. G. (1974*b*) Inhibitors of haemopoietic cell proliferation: Reversibility of action. *Br. J. Cancer*, **29**, 407.

Lord, B. I., Shah, G. P. & Lajtha, L. G. (1977) The effects of red blood cell extracts on the proliferation of erythrocyte precursor cells, *in vivo. Cell Tissue Kinet.*, **10**, 215.

Rytömaa, T. & Kiviniemi, K. (1968) Controls of DNA duplication by means of the granulocyte chalone. *Eur. J. Cancer*, **4**, 595.